# Author's reply to the letter entitled “Critique of omnipolar mapping claims in superior vena cava isolation: A call for standardization”

**DOI:** 10.1002/joa3.70082

**Published:** 2025-04-30

**Authors:** Naoto Oguri, Yousaku Okubo, Takehito Tokuyama, Noboru Oda, Yukiko Nakano

**Affiliations:** ^1^ Department of Cardiovascular Medicine, Graduate School of Biomedical and Health Sciences Hiroshima University Hiroshima Japan

Dear Editor,

We appreciate the insightful critique by Khawar et al. regarding our study, “Novel Omnipolar Mapping Technology for Effective Superior Vena Cava Isolation: A Randomized Clinical Trial.”^1^ Their engagement in this discourse underscores the importance of methodological rigor and transparency in the advancement of electrophysiology.

Regarding the concerns raised about procedural criteria and the relevance of reduced radiofrequency (RF) applications, our study followed a standardized protocol informed by previous literature and clinical experience. Although we acknowledge that the number of RF applications in our study may appear higher than some historical averages, as discussed in our original manuscript, the use of circumferential isolation—as opposed to a selective point‐by‐point ablation strategy targeting sequential ablation of fast potential sites in the superior vena cava (SVC)—likely contributed to the observed differences in RF application numbers and overall procedure times. Previous studies using high‐density mapping for SVC isolation have reported that successful isolation requires an average of 10–15 RF applications.^2^, ^3^, ^4^, ^5^ Our findings are consistent with these reported values.

Operator variability is indeed a critical factor in electrophysiological studies. In this study, all procedures were performed by four experienced electrophysiologists, and the cases were evenly distributed among the operators across the groups. In the omnipolar mapping technology (OT) group, operators A, B, C, and D performed 4, 9, 7, and 5 cases, respectively. In the conventional method (CM) group, operators A, B, C, and D performed 8, 9, 5, and 3 cases, respectively. Accordingly, no significant difference was found in the distribution of cases performed (*p* = .54). Although this study is a randomized controlled trial, it is an open‐label study; thus, the possibility of bias due to unboundedness cannot be excluded.

In terms of sinoatrial node (SN) localization, we acknowledge that external validation is lacking. As noted, this study did not include a formal validation method or employ additional modalities to confirm the SN location. Therefore, although our observations suggest that OT can help identify the SN in cases in which bipolar mapping fails, any potential advantage of OT remains speculative without standardized or external validation.

In this study, the color contour was configured to display 15 distinct colors, each representing a 10 ms interval, to enhance the delineation of the right atrium (RA)–SVC conduction block line. Despite the relatively high number of color bands, the conduction block line was still defined based on an activation delay of ≥30 ms, which is consistent with the criteria used in previous studies.^2^ Thus, although the original method was used, the fundamental definition of the conduction block remains unchanged.

We appreciate the reviewer's insightful comment regarding the limitations associated with the use of proprietary technology and software. The lack of external validation limits the generalizability of our findings. However, conducting simultaneous or sequential mapping using multiple systems within the same patient is not feasible in a clinical setting because of procedural and technical constraints. Consequently, external validation using alternative mapping platforms was not performed.

In conclusion, although we recognize the points raised by Khawar et al., we believe that our study offers valuable insights into the role of omnipolar mapping in SVC isolation. Although these findings may not be immediately generalizable, OT appears to offer substantial advantages over the anatomical approach because of the lower number of RF applications and shorter procedural time.

## FUNDING INFORMATION

The authors did not receive any specific funding for this study.

## CONFLICT OF INTEREST STATEMENT

Authors declare no conflict of interests for this article.

## APPROVAL OF THE RESEARCH PROTOCOL

This study was approved by the Institutional Ethics Committee of Hiroshima University's Graduate School of Biomedical Science (Approval No. C2021‐0339, registered on September 30, 2020), and was conducted in strict adherence to the principles outlined in the Declaration of Helsinki.

## INFORMED CONSENT

All participants provided written informed consent.
